# Endangered Cultus Lake sockeye salmon exhibit genomic evidence of hypoxic and thermal stresses while rearing in degrading freshwater lacustrine critical habitat

**DOI:** 10.1093/conphys/coab089

**Published:** 2021-11-25

**Authors:** Arash Akbarzadeh, Daniel T Selbie, Lucas B Pon, Kristina M Miller

**Affiliations:** 1 Fisheries and Oceans Canada, Pacific Biological Station, 3190 Hammond Bay Road, Nanaimo, British Columbia, V9T 6N7, Canada; 2 Department of Fisheries, Faculty of Marine Science and Technology, University of Hormozgan, 9th km of Minab Road, Bandar Abbas, 79161 93145, Iran; 3 Fisheries and Oceans Canada, Science Branch, Pacific Region, Cultus Lake Salmon Research Laboratory, 4222 Columbia Valley Hwy, Cultus Lake, British Columbia, V2R 5B6, Canada

**Keywords:** thermal stress, sockeye salmon, pathogen, hypoxia, Cultus Lake, Biomarker

## Abstract

Water quality degradation due to lake eutrophication and climate change contributes to the risk of extirpation for the endangered Cultus Lake sockeye salmon. Sockeye salmon juveniles experience both low-oxygen water in profundal lake habitats and elevated temperatures above the thermocline during diel vertical migrations in summer and fall when the lake is thermally stratified. We used a transcriptomic tool (Salmon Fit-Chip) to determine whether salmon were experiencing thermal and/or hypoxic stress during this period. The results showed that over one-third of the fish were responding to either hypoxic (35.5%) or thermal stress (40.9%) during periods when these environmental stressors were pronounced within the lake, but not during periods when profundal dissolved oxygen was elevated and the water column was isothermal and cool. The most consistent signs of hypoxic stress occurred during July (52.2%) and September (44.4%). A total of 25.7% of individual fish sampled during months when both stressors were occurring (July, September, October) showed signatures of both stressors. When a combination of hypoxic and thermal stress biomarkers was applied, 92% of fish showed evidence of one or both stressors; hence, for at least several months of the year, most sockeye salmon juveniles in Cultus Lake are experiencing anthropogenically environmentally induced stress. We also detected the presence of pathogenic ciliate *Ichthyoptherius multifiliis* in the gill tissue of juveniles, with a higher infection signal in Cultus Lake compared to juveniles from nearby Chilliwack Lake. These data provide powerful new evidence that Cultus Lake sockeye salmon, which experience relatively lower juvenile survival than Chilliwack sockeye salmon, are more compromised by stress and carry a higher level of infection of at least one pathogenic agent. Thus, we hypothesize that the cumulative or synergistic interplay between stressors and diseases, clearly documented to be occurring within Cultus Lake, are contributing to increased mortality of endangered sockeye salmon.

## Teaser

Using a transcriptomic tool (Salmon Fit-Chip), we provided powerful new evidence that Cultus Lake sockeye salmon juveniles experience thermal and hypoxic stress during the period of low-oxygen water in profundal lake habitats and elevated temperatures above the thermocline in summer and fall when the lake is thermally stratified. The cumulative or synergistic interplay between stressors and diseases, clearly documented to be occurring within Cultus Lake, are contributing to increased mortality of endangered sockeye salmon. Eutrophication and hypolimnetic dissolved oxygen depletion, which is exacerbated by climate change, will increase the risk of extirpation for sockeye salmon in Cultus Lake.

## Introduction

The Fraser River watershed produces the most abundant bilateral (the USA and Canada) sockeye salmon (*Oncorhynchus nerka*) fisheries opportunities of any drainage in North America, contributing significantly to commercial, recreational and Indigenous interests. Within the watershed, variation among populations in run timing, abundance status and trends (i.e. cyclical dominance) and other life history traits evoke a complex fisheries management framework (DFO, 2020a). As Fraser River sockeye salmon harvests are managed in a mixed-stock fisheries context (common migration run timings across certain stocks), less abundant conservation units (CUs) can have disproportionate influences on aggregate fishery exploitation potentials and can commensurately experience abundance reductions in mixed-stock fisheries.

The Cultus Lake sockeye salmon stock, one of the most intensively studied populations of salmon in British Columbia, Canada, has experienced a dramatic decline in the abundance of returning adults over the past 40–50 years (Schubert *et al.*, 2002; COSEWIC, 2003). Several factors, including harvest and predation, poor ocean survival, diseases and parasites and exposure to warmer temperatures, have been identified as contributing to the collapse of the stock (Schubert *et al.*, 2002; COSEWIC, 2003). In response, Cultus Lake sockeye salmon were emergency listed as endangered by the Committee on the Status of Endangered Wildlife in Canada in 2002, an assessment that was confirmed in 2003 (COSEWIC, 2003) and upheld during reassessment in 2017 (COSEWIC, 2017). Cultus Lake sockeye salmon are classified as part of the Fraser River sockeye salmon late-run timing group, which have historically migrated upriver in the fall after holding in the Straight of Georgia for up to 6 weeks (Burgner, 1991).

Cultus sockeye salmon adults co-migrate up the Fraser River with numerically abundant CUs, such as the Shuswap Complex-L CU, which has experienced record escapements in recent years (dominant line), and commensurate fisheries. The conservation status of Cultus Lake sockeye salmon necessitated significant reductions in exploitation rates on other late-run sockeye salmon stocks in the Fraser River throughout the late 1990s and 2000s. These stocks experienced a historical dominant cycle average exploitation of ~70% (1954–1994), which was reduced to ~32% (1998–2011) in support of the recovery of the Cultus Lake CU, despite significant increases in Late Shuswap stock production, which likely explain recent dominant line density-dependent survival limitations in freshwater (i.e. rearing capacity limitation; Grant *et al.*, 2011), eroding future production and fisheries opportunities.

Incidental harvest on the Cultus Lake sockeye salmon CU has subsequently increased, despite persistently low returns and smolt outputs, directly pitting conservation objectives against current and future fisheries opportunities (DFO, 2018). Conservation hatchery efforts have likely staved off extirpation to date (Bradford *et al.*, 2010), but overexploitation in the mixed-stock fishery continues to be a primary driver of its endangerment (COSEWIC, 2003; Cultus Sockeye Recovery Team, 2009). Recent limnological and fisheries investigations on Cultus Lake by Department of Fisheries and Oceans (DFO)’s Lakes Research Program have revealed substantial in-lake mortality of juveniles, linked to lake eutrophication (artificial nutrient enrichment) and the interactive influences of climate variability and change (Putt *et al.*, 2019; DFO, 2020b). Freshwater survival is strongly related to deep water dissolved oxygen (DO) concentrations, which are depleted with enhanced lake stratification, intensifying lake productivity and aerobic decomposition of excess organic matter, arising from the interactions between eutrophication and climate change (Shortreed, 2007; Putt *et al.*, 2019; DFO, 2020b). This new linkage is the first demonstration of a lake habitat effect on Cultus Lake sockeye salmon survival, and owing to the direct linkages to eutrophication, likely reversible with appropriate watershed and airshed nutrient management (inputs already modelled; Putt *et al.*, 2019). However, several oxygen-mediated pathways of effect on sockeye salmon juvenile survival exist, which could be the ultimate drivers of in-lake mortality, including direct hypoxic stresses, increased susceptibility to diseases or pathogens and exposure to internal loading of contaminants (e.g. ammonia, metals) from lake sediments. Determination of the ultimate mechanism(s) of influence on juvenile survival is critically important to engage informed habitat and fisheries management that succeeds in rebuilding this stock and alleviating constraints targeting other abundant late-run CUs in the Fraser River mixed-stock fishery.

Shortreed (2007) compared limnological data taken in the 1920s–1930s with those from 2001–2003 and found that both biological productivity and surface water temperatures had increased through the 20th century, highlighting anthropogenic nutrient loading (i.e. cultural eutrophication) and climate change as the most parsimonious forcings. Lake ecosystem degradation associated with eutrophication is exacerbated by climate change (Moss *et al.*, 2011). Warmer air temperatures and potentially reduced summer precipitation yield stronger and protracted lake stratification in Cultus Lake, enhancing algal production and lengthening the period of hypolimnetic separation from atmospheric oxygen recharge (Sumka, 2017). Lake epilimnion warming and enhanced hypolimnetic DO depletion could create a ‘temperature-oxygen squeeze’ whereby increasingly hypoxic hypolimnetic waters and thermally sub-lethal to lethal epilimnetic waters encroach upon one another, reducing and degrading available rearing habitat for Cultus Lake sockeye salmon (Putt *et al.*, 2019; Kerker, 2020). Therefore, eutrophication and hypolimnetic DO depletion increase the risk of extirpation for Cultus Lake sockeye salmon that requires profundal and benthic habitats (DFO, 2020b).

Measuring DO concentrations in aquatic ecosystems is one aspect of monitoring hypoxic stress for biota. However, frequent DO measurements over large regions and long periods of time are often impractical. Furthermore, such measurements do not directly address whether hypoxic stress was experienced by fish. Therefore, the use of biomarkers indicating fish responses to stressors is an important approach (Zhang *et al.*, 2012) and a more integrative technique (Froehlich *et al.*, 2015). The ideal biomarker(s) should be specific to the stressor of interest, be easy to assay and be relatively unaffected by sampling procedures (Zhang *et al.*, 2012). One major advantage of a biomarker is the ability to detect sub-lethal impacts at low levels of stressor intensity. Molecular ecologists have become interested in studying early genetic responses of various organisms to stressors such as hypoxia (Nikinmaa and Rees, 2005; Boswell *et al.*, 2009).

A genomic tool called the ‘Salmon Fit-Chip’ has been recently developed by the DFO Molecular Genetics Laboratory to identify specific stressors (e.g. thermal, hypoxic, osmotic and general stress) and diseases (infectious agents, viral disease, inflammation, immune stimulation) in salmon and trout that is based on targeted host response profiling of gill tissue (Akbarzadeh *et al.*, 2018, 2020; Miller *et al.*, 2016, 2017; Houde *et al.*, 2019). Gill is an ideal tissue to monitor environmental responses because of its direct contact with water and can be sampled non-lethally. The Salmon Fit-Chip is a microfluidics quantitative (qRT)-PCR chip that can simultaneously assess 96 gene assays in 96 samples at once. The chip is populated with biomarker panels that when co-activated are predictive of the presence of specific stressors that have been validated in a series of control challenge studies. To predict the presence of a specific stressor requires only 6–12 co-expressed biomarkers (Miller *et al.*, 2017; Houde *et al.*, 2019). This is the first tool of its kind to enable the simultaneous assessment of multiple stressors and disease influences on salmonids, or any other species. We paired this new genomic and transcriptomic tool with our habitat and fish condition information in Cultus Lake (and Chilliwack Lake as a reference) to assess fish stress responses to these potential drivers of poor freshwater juvenile survival. Ultimately, identifying the underlying causes of juvenile mortality in nursery habitats will actively inform habitat and fisheries management on remedial actions to improve Cultus Lake sockeye salmon survival, with the goals of ensuring the genetic lineage of this distinct CU and alleviating future constraints on the broader Fraser River late-run sockeye salmon fisheries.

## Material and methods

### Fish collection

**Fig. 1:**
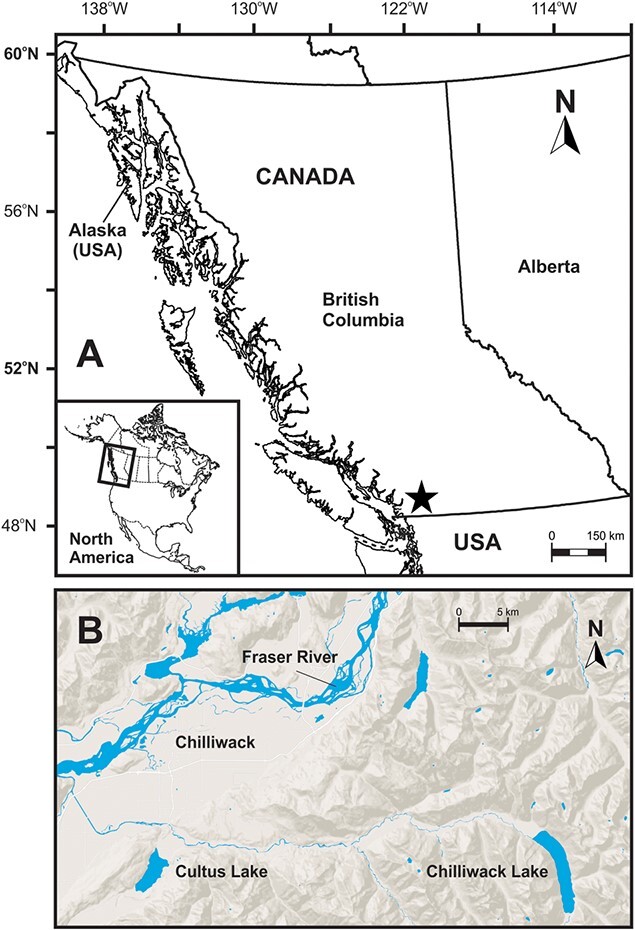
Map of Cultus Lake and Chilliwack Lake in British Columbia, Canada.

Juvenile sockeye salmon were collected during acoustic-trawl surveys (MacLellan and Hume, 2010) of Cultus (49.0679° N, 121.9762° W) and Chilliwack lakes (49.0576° N, 121.4143° W) British Columbia, Canada (Fig. 1). In Cultus lake, six sampling events occurred in July, September, October and November 2018 and in March and April 2019, while sockeye salmon in Chilliwack lake were sampled in August and November 2018. Optimal water column trawling depths for sockeye salmon juveniles were determined from hydroacoustic echogram data collected with a Biosonics model DT-X echosounder with a split-beam transducer (208 kHz). As fish densities were relatively low, the trawl was set to a water depth that maximized fishing while maintaining a safe operating distance from the bottom of the lake. All work was conducted at night as sockeye salmon juveniles exhibit strong diel migration behaviour (Levy, 1989) and are only within range of the hydroacoustic system and trawl at this time (Burczynski and Johnson, 1986), occupying deeper, potentially more hypoxic waters during the day.

Trawls were conducted from a 7-m vessel, equipped with a hydraulic crane that allowed controlled setting, opening and closure and retrieval of the net. An 18-m-long midwater trawl with an opening measuring 3 m in width by 7 m in depth was used to collect fish from the study lakes. The trawl net was constructed from a 4-inch stretched mesh near the opening and decreased in size across five steps (4″, 2″, 1″, 3/4″, 1/2″, 1/8″) to 1/8 inches bobbin near the polyvinyl chloride (PVC) cod end, affixed with a screw cap to permit allows rapid fish retrieval and minimization of turbulent flow and holding impacts on caught fish. Trawl durations and depths are summarized in Table 1.

**Table 1:** Depth and duration of all trawls conducted in Cultus Lake and Chilliwack Lake and the fish number and size (mean ± standard deviation)

Sample interval	Lake	Date	Trawl ID	Duration (min)	Depth (m)	Number of fish	Fork length (mm)	Condition
**1**	Cultus	10 July 2018	20 180 201	35	16	24	52.3 ± 18.2	HP^a^-WE^b^
**2**	Chilliwack	14 August 2018	20 180 301	40	18	11	61.3 ± 12.1	NP^c^-WE
**2**	Chilliwack	14 August 2018	20 180 302	40	19	4	66.6 ± 11.8	NP-WE
**3**	Cultus	04 September 2018	20 180 401	40	18	16	63.6 ± 4.1	HP-WE
**3**	Cultus	04 September 2018	20 180 402	40	18	14	59.8 ± 6.5	HP-WE
**4**	Cultus	04 October 2018	20 180 801	39	25	30	69.3 ± 5.9	HP-WE
**5**	Chilliwack	01 November 2018	20 181 001	40	21	8	63.0 ± 7.9	NP-CT^d^
**5**	Chilliwack	01 November 2018	20 181 002	40	21	8	67.0 ± 7.2	NP-CT
**6**	Cultus	06 November 2018	20 181 101	40	26	19	73.6 ± 5.0	HP-CT
**7**	Cultus	06 March 2019	20 190 101	30	28	22	94.1 ± 10.7	NP-CT
**8**	Cultus	04 April 2019	20 190 201	28	29	8	106.0 ± 9.5	NP-CT
**8**	Cultus	04 April 2019	20 190 202	40	29	7	96.6 ± 14.5	NP-CT

a ^a^HP, for periods of hypoxia at the bottom of the lake.

b ^b^WE, for months with warm temperatures above the epilimnion of the lake.

c ^c^NP, for periods of normoxia at the bottom of the lake.

d ^d^CT, for months with lower water column temperatures of the lake.

Once the catch was brought on board, fish were quickly transferred to a 20 -l bucket containing fresh lake water. Sockeye salmon juveniles were then euthanized in another bucket containing a solution of MS-222 (tricane methanesulfonate, 250 ppm) buffered with sodium bicarbonate. Fish were observed to ensure euthanasia took place in a rapid manner (i.e. cessation of gill ventialization). Whole fish were measured onboard the boat, then immediately frozen in liquid nitrogen and stored at −80°C in a freezer at the Cultus Lake Salmon Research Laboratory, and subsequently the Molecular Genetics Laboratory, Pacific Biological Station, Nanaimo, British Columbia, Canada, until used for RNA extraction. In the laboratory, tissues including gill, muscle, liver, heart, kidney and spleen were dissected from each frozen fish. Tools were disinfected between samples using 3–5 min of 10% bleach and immersion in 95% ethanol and a flame, being allowed to cool before use on the next sample. A total of 50–100 mg of dissected gill tissue was used for RNA extraction. Other dissected tissues were archived in a −80°C freezer to serve in various other types of studies.

### Limnology

Water temperatures and DO concentrations from the water surface to just above the lake bottom were measured with a YSI ProODO oxygen metre (YSI Inc., Yellow Springs, OH, USA) on each sampling month before the acoustic-trawl survey. Vertical profiles were obtained from two established DFO Lakes Research Program monitoring stations on Cultus Lake (DFO limnology stations 1 and 6).

### Gene expression

Our previous validation of hypoxia biomarkers performed using a stress-challenge study in juvenile Chinook salmon (*Oncorhynchus tshawytscha*) showed that the combinations of biomarkers most strongly differentiating normoxic and hypoxic states varied between salinity environments. Hence, in this study, to identify signals of hypoxia in fish using the freshwater lake habitat, we combined seven hypoxia genes (biomarkers) that previously showed the strongest differential activity for fish exposed to hypoxic conditions in freshwater (Akbarzadeh *et al.*, 2020) with four general hypoxia biomarkers from the literature (Houde *et al.*, 2019) and applied these to the juvenile sockeye salmon samples obtained from Cultus and Chilliwack lakes (Table 2). To examine thermal stress, 10 biomarkers previously validated in salmonids (Akbarzadeh *et al.*, 2018; Houde *et al.*, 2019) were applied (Table 2).

**Table 2:** Biomarkers used to identify hypoxia and thermal stress signals in Cultus Lake sockeye salmon

Gene name	Assay name
Hypoxia biomarkers	
Anillin-like	Anillin
Aurora kinase B-like	AURKB
Kinesin-like protein KIF2C	KIF2C
Non-SMC condensin II complex subunit D3	Ncapd3
Rho GTPase-activating protein 11A-like	ARHGAP11A
NDC80, kinetochore complex component	Ndc80
Chromosome-associated kinesin KIF4-like	Kif4
Fructose-bisphosphate aldolase A1	ALD_4._v1
Neuroglobin	Ngb1_2
Haemoglobin	HemA1_1
Glycogen phosphorylase	GlPh_1
Thermal biomarkers	
Elongation factor 2	Ef2_14
FK506-binding protein 10 precursor	FK506_19_v2
FK506-binding protein 10 precursor	FK506_3&6 _v2
Heat shock 70 kDa protein	Hsp70_6
Heat shock protein 90 alpha	Hsp90a_15_v2
Heat shock protein 90 alpha like	HSP90alike_6
Mitogen-activated protein kinase kinase kinase 14	Map3k14_3
Serpin H1 precursor (HSP47)	SERPIN_20_v1
Serpin H1 precursor (HSP47)	SERPIN_9
Splicing factor, arginine/serine-rich 2	SFRS2_3_v2

We also applied assays to six freshwater infectious agents, including *Ichthyophthirius multifiliis*, *Loma salmonae, Ceratonova shasta*, *Parvicapsula minibicornis*, *Syngnamydia salmonisa* and Pacific Salmon nidovirus (PsNV), that are commonly reported in sockeye salmon gill tissues in BC lakes (Nekouei *et al.*, 2018; note the latter virus is newly discovered; Mordecai *et al.*, 2019).

A total of 155 individuals were examined for gill gene expression, distributed between months with and without each stressor and between Cultus Lake and the control system, Chilliwack Lake, for which there is no evidence of hypoxia or substantially elevated water surface temperatures (see Table 1 for details). The methods used for gill tissue homogenization, RNA extraction and quantification and cDNA synthesis have been previously described (Akbarzadeh *et al.*, 2018; Houde *et al.*, 2019).

To test the efficiency of the examined biomarkers, cDNA from RNA extractions of pooled gill tissues from all individuals were serially diluted from 1/5 to 1/625 in five dilutions. Specific target amplification (STA) was performed to enrich targeted sequences within the pools using 3.76 μl 1X TaqMan PreAmp master mix (Applied Biosystems), 0.2 μM of each of the primers and 1.24 μl of cDNA, according to Fluidigm protocols and as previously described (Akbarzadeh *et al.*, 2018; Houde *et al.*, 2019). Samples were run on a 14-cycle Polymerase Chain Reaction (PCR) program, with excess primers removed with EXO-SAP-IT (Affymetrix), and diluted 1/5 in DNA suspension buffer. The diluted samples and assays were run in one technical replication, except for the pathogens that were run in duplicate. Each run contained additional negative processing controls and serial dilutions of pathogen standards, as described in Miller *et al.* (2016). For sample reactions, 3.0 μl 2X TaqMan mastermix (Life Technologies), 0.3 μl 20X GE sample loading reagent and 2.7 μl STA product were used. For assay reactions, 3.3 μl 2X assay loading reagent, 0.7 μl DNA suspension buffer, 1.08 μl forward and reverse primers (50 uM) and 1.2 μl probe (10 μM) were used. The PCR was run at 50°C for 2 min, 95°C for 10 min, followed by 40 cycles of 95°C for 15 s and then 60°C for 1 min. Data were extracted using the Real-Time PCR Analysis Software (Fluidigm) using Ct thresholds set manually for each assay. PCR efficiencies for each assay were calculated using (10 ^1/slope^ − 1) × 100, for which the slope was estimated by plotting the Ct over the serial dilutions of cDNA.

The 96.96 gene expression dynamic array (Fluidigm Corporation, CA, USA) was applied and generally followed by Miller *et al.* (2016). qRT-PCR data were analysed with Real-Time PCR Analysis 3 Software (Fluidigm Corporation, CA, USA). Expression of the target genes was normalized to the expression of the housekeeping gene, S100 calcium-binding protein (78d16.1), which was found to be the most suitable tested housekeeping gene in NormFinder analysis, as previously described (Houde *et al.*, 2019). Sample gene expression was normalized with the ∆∆Ct method (Livak and Schmittgen, 2001) using the inter-array calibrator sample (undiluted pooled sample). Gene expression was then log transformed: log_2_ (2^-∆∆Ct^).

**Fig. 2:**
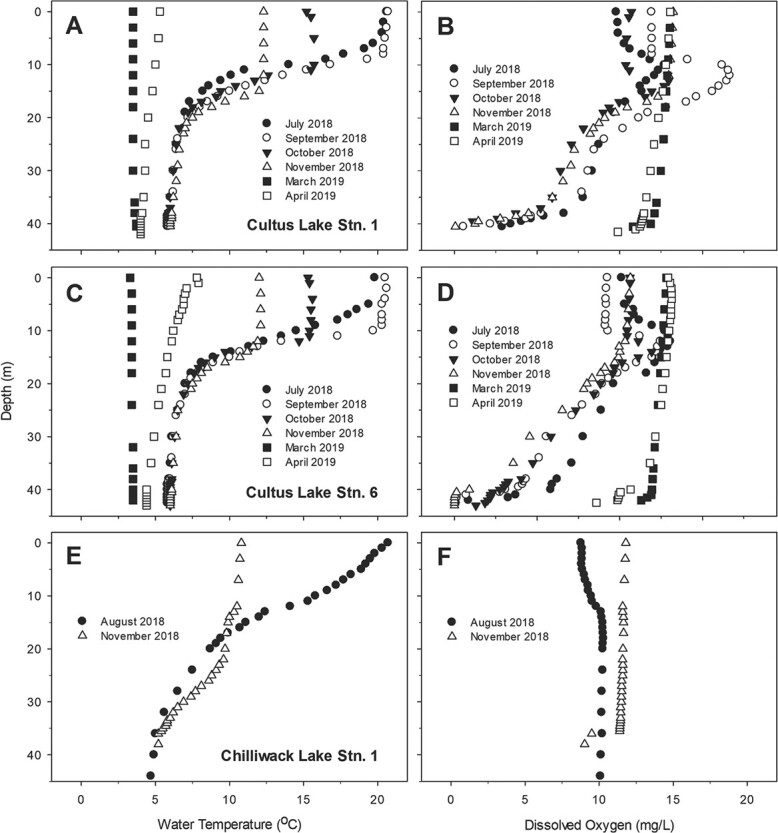
Vertical temperature and dissolved oxygen (DO) profiles in Cultus Lake in July, November, October, November 2018, March and April 2019, and Chilliwack Lake in August and November 2018. A) Cultus Lake temperature station 1; B) Cultus Lake DO station 1; C) Cultus Lake temperature station 6; D) Cultus Lake DO station 6; E) Chilliwack Lake temperature; F) Chilliwack Lake DO.

### Data analysis

Analyses were performed using R 3.4.4 (R Core Team) at a significance level of α = 0.05. The hypoxic and thermal stress biomarkers were subjected to a principal components analysis (PCA) using the *fviz_pca* function of the *factoextra* R package (Kassambara and Mundt, 2017). This function provided 95% confidence ellipses for groups within each data set. Then, the entire dataset was divided into an analytical training set (two-thirds) and an analytical testing set (one-third) for testing the classification ability. The classification ability of the groups was also examined in the training set by subjecting the hypoxia and thermal biomarkers to linear discriminant analysis (LDA) followed by a determination of classification performance on the testing set.

Differences in prevalance of *I. multifiliis* in fish sampled during months with hypoxic profundal (HP) waters and normoxic waters and during months with warm epilimnetic waters and lower water column temperatures were analysed by a Student *t*-test (*P* < 0.05).

## Results

### Limnology

The physical structure of both study lakes changed with seasonality through the course of our study. Cultus Lake was thermally stratified for all fish sampling events from July to November. Epilimnetic temperatures exceeded 20°C in July and September. Water surface temperatures did not exceed 12°C in November. The water column was nearly isothermal in March, with surface heat absorption evident by April (Fig. 2A and C). Chilliwack Lake was also thermally stratified during the two sampling events in August and November. The Epilimnetic temperature exceeded 20°C in August, whereas it was below 11°C in November (Fig. 2E).

**Fig. 3:**
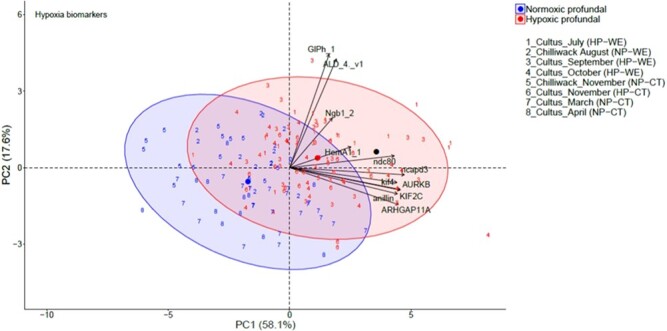
Canonical plots of the first two principal components of the hypoxia biomarkers for Sockeye Salmon in Cultus Lake and Chilliwack Lake from July 2018 to April 2019. Principal component analysis (PCA) was performed on the entire dataset. Ellipses represent 95% confidence areas for the groups within treatments. Centroids are represented by the largest point of the same colour. Arrows represent loading vectors of the biomarkers. HP: hypoxic profundal (for periods of hypoxia at the bottom of the lake), WE: warm epilimnion (for months with warm temperatures above the epilimnion of the lake), normoxic profundal (For periods of normoxia at the bottom of the lake), CT: cool temperature (for months with lower water column temperatures of the lake).

DO concentrations dramatically declined with the water depth below the metalimnion of Cultus Lake, reaching hypoxic levels in July, September, October and November (Fig. 2B and D). Hypolimnetic DO drop to below 5 mg/l in water depths 35–43 m during these months, reaching near-anoxia in the profundal zone (Fig. 2D). In March and April, the water column of Cultus Lake was highly oxygenated, and epilimnetic and hypolimnetic DO concentrations similar, owing to lake mixes (Fig. 2B and D). In contrast with Cultus Lake, DO concentrations in Chilliwack Lake exceeded 9 mg/l over the top 50 m of the water column in both summer (August) and fall (November) (Fig. 2F).

Here, we use the terms ‘warm epilimnion’ (WE) for months with WE temperatures (July, August, September and October), ‘hypoxic profundal’ for periods of hypoxia in the profundal zone (July, September, October, and November), ‘cool temperature’ (CT) for months with lower temperatures in the whole water column owing to lake mixis (November, March and April) and ‘normoxic profundal’ (NP) for periods of NP waters (March and April in Cultus Lake; August and November in Chilliwack Lake).

### Gene expression

The gene expression profiles for the 11 hypoxic and 10 thermal stress biomarkers applied across all 155 fish caught from Cultus Lake and Chilliwack Lake from July 2018 to April 2019 are presented in the Appendix 1.

### Hypoxia biomarkers

PCA of our hypoxia biomarkers exhibited clear ordinal separation along PCA Axis 1, the main direction of variation in the transcriptomic data, for juvenile sockeye salmon sampled in Cultus Lake during months with HP waters versus those with NP conditions and versus those sampled under normoxic conditions in Chilliwack Lake. Some ordinal separation was also observed along PCA Axis 2 among fish sampled during months with warm surface waters, versus those captured during cooler water temperatures (Fig. 3). From the 93 fish sampled during months with hypoxic conditions at depth, 33 (35.5%) fish clustered outside the ellipse boundaries of the fish sampled during months of normoxia. None of the 62 fish sampled during periods of profundal normoxia in Cultus Lake and water column normoxic conditions in Chilliwack Lake were clustered in the ellipse boundaries of the fish sampled during profundal hypoxia in Cultus Lake. The most consistent signs of hypoxic stress were evident in July (52.2%) and September (44.4%). The lowest signs of hypoxic stress were observed among fish sampled when Cultus Lake surface temperatures had cooled in November (15.8%), but still exhibited HP waters (Fig. 3). The mean LDA classification ability for HP conditions and normoxic normoxic waters was 78.3% (Table 3).

**Table 3:** LDA classification ability of the groups using the hypoxia, thermal and combination of hypoxia and thermal biomarkers

	Hypoxia biomarkers		Thermal biomarkers			Hypoxia + thermal biomarkers				
	HP	NP		WE	CT		HP^a^-WE^b^	NP^c^-WE	NP-CT^d^	HP-CT
HP	16	4	WE	19	3	HP-WE	21	2	0	1
NP	7	23	CT	2	26	NP-WE	1	3	0	0
						NP-CT	0	0	15	1
						HP-CT	0	0	0	6

LDA training set used two-thirds of the entire dataset. Presented are the classifications on the remaining one-third testing set. Columns are the LDA classification groups and rows are the real group. Columns are the LDA classification groups and rows are the real group.

a ^a^HP, for periods of hypoxia at the bottom of the lake.

b ^b^WE, for months with warm temperatures above the epilimnion of the lake.

c ^c^NP, for periods of normoxia at the bottom of the lake.

d ^d^CT, for months with lower water column temperatures of the lake.

**Fig. 4:**
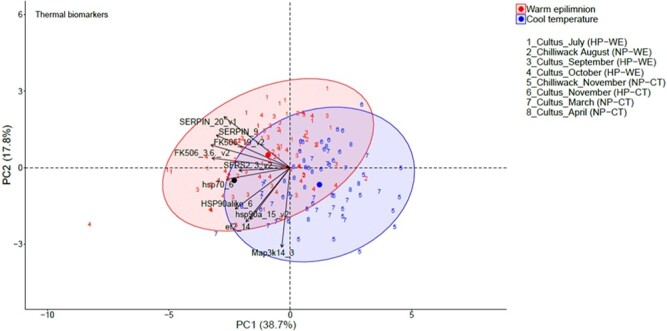
Canonical plots of the first two principal components of the thermal biomarkers in Cultus Lake and Chilliwack Lake from July 2018 to April 2019. Principal component analysis (PCA) was performed the entire dataset. Ellipses represent 95% confidence areas for the groups within treatments. Centroids are represented by the largest point of the same colour. Arrows represent loading vectors of the biomarkers. HP: hypoxic profundal (for periods of hypoxia at the bottom of the lake), WE: warm epilimnion (for months with warm temperatures above the epilimnion of the lake), normoxic profundal (For periods of normoxia at the bottom of the lake), CT: cool temperature (for months with lower water column temperatures of the lake).

### Thermal biomarkers

PCA of our thermal biomarkers exhibited PCA Axis 1 ordinal separation of juvenile sockeye salmon sampled in warm months versus those captured during periods of cooler water column temperatures and some ordinal separation along PCA Axis 2 among fish sampled during months with HP in Cultus Lake versus those captured during months with a normoxic water column (Fig. 4). From 88 fish sampled during months with warm water epilimnetic temperatures and hypoxic conditions at depth, 36 (40.9%) fish fell within the thermal-responsive cluster and outside of the ordinal boundaries of fish sampled during months of cooler water temperatures. The most consistent signs of thermal stress were observed during July (52.2%), October (45.8%) and September (37.0%). The mean LDA classification ability for months with warm epilimnetic temperatures and months with cool water column temperatures was 89.6% (Table 3).

### Overall classification

**Fig. 5:**
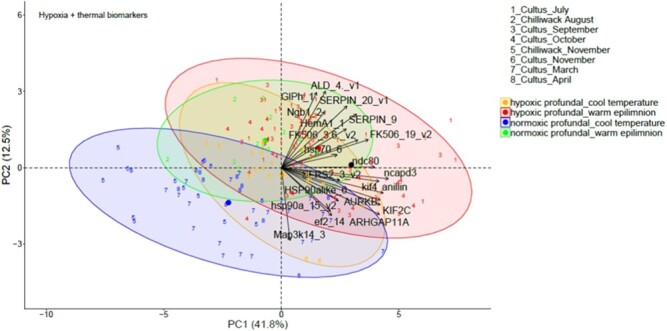
Canonical plots of the first two principal components of both hypoxia and thermal biomarkers in Cultus Lake and Chilliwack Lake from July 2018 to April 2019. Principal component analysis (PCA) was performed the entire dataset. Ellipses represent 95% confidence areas for the groups within treatments. Centroids are represented by the largest point of the same colour. Arrows represent loading vectors of the biomarkers.

PCA using both hypoxia and thermal biomarkers indicates the strongest ordinal separation among juvenile sockeye salmon sampled in months with HP waters and warm surface waters versus those from NP waters and cooler water temperatures (Fig. 5). From 74 fish sampled during months with warm surface waters and hypoxic conditions at depth, 68 (91.9%) fish were contained within the hypoxia-thermal-responsive cluster, with the remaining 8.1% of individuals overlapping physiologically with fish sampled during months of profundal normoxia and low water temperatures (Fig. 5). Moreover, there was a high degree of ordinal separation along PCA Axis 2, among fish sampled in Chilliwack Lake in August (normoxic, WE temperatures) and those sampled during normoxic, cooler water column temperatures. From 14 fish sampled in normoxic and warm water temperatures, 11 (78.6%) fish were contained in the normoxic, thermal-responsive cluster. The average LDA classification ability for all four different conditions, i.e. WE-HP, CT-NP, WE-NP and CT-HP, was 89.1% (Table 3).

From 74 fish sampled in both warm and hypoxic months in Cultus Lake (July, September and October) 19 (25.7%) fish exhibited signals of both hypoxia and thermal stress when analysed using either hypoxic and thermal stress biomarkers separately in PCA. Moreover, 26 (35.1%) fish demonstrated no genomic signature of hypoxic or thermal stress.

### Pathogen biomarkers

Among the six pathogen biomarkers tested in our study, only *I. multifiliis* infestation was detected in sockeye salmon juveniles. In Cultus Lake, *I. multifiliis* was detected in 89 (70.1%) of 127 fish. In Chilliwack Lake, *I. multifiliis* was detected in 10 (32.3%) of 31 individuals. The proportion of *I. multifiliis* detections in fish within the study lakes varied considerably between months and were most elevated in Cultus Lake in September (96.4%), November (100%) and April (91.7%) and in November (60%) in Chilliwack Lake (Table 4). The prevalence of *I. multifiliis* in fish sampled during months with HP waters in Cultus Lake (71.3%) was significantly higher (*P* < 0.05) than in fish sampled in normoxic waters across both lakes (53.2%). However, the prevalence of *I. multifiliis* in fish sampled during months with warm epilimnetic waters (61.3%) was not significantly different (*P* > 0.05) from fish captured during months with lower water column temperatures (63.2%).

**Table 4:** Prevalence of infectious agent detections in sampled fish

Pathogen	Cultus, July	Chilliwack, August	Cultus, September	Cultus, October	Chilliwack, November	Cultus, November	Cultus, March	Cultus, April
*I. multifiliis*	13.0	60.0	96.4	75.0	6.7	100.0	54.5	91.7
*Loma salmonae*	0.0	0.0	0.0	0.0	0.0	0.0	0.0	0.0
*Ceratonova shasta*	0.0	0.0	0.0	0.0	0.0	0.0	0.0	0.0
*Parvicapsula minibicornis*	0.0	0.0	0.0	0.0	0.0	0.0	0.0	0.0
*Syngnamydia salmonisa*	0.0	0.0	0.0	0.0	0.0	0.0	0.0	0.0
Pacific salmon nidovirus	0.0	0.0	0.0	0.0	0.0	0.0	0.0	0.0

Almost all different conditions, i.e. hypoxic warm temperature (Cultus Lake in September and October), hypoxic normal temperature (Cultus Lake in November), normoxic warm temperature (Chilliwack Lake in August) and normoxic normal temperature (Cultus Lake in April), showed elevated signals of gill *I. multifiliis* infection. In Chilliwack Lake, the prevalence of gill *I. multifiliis* in summer month (August) with high water temperatures in the epilimnion was remarkably higher than that of fall month (November) with cold water column temperatures (Table 4). Moreover, the percentage of the fish that showed detection of infectious *I. multifiliis* in Cultus Lake in November (100.0%) under HP condition, was obviously higher than that of the normoxic Chilliwack Lake (6.7%) during the same time and at similar water column temperatures (Table 4).

## Discussion

Cultus Lake serves as critical habitat for the endangered Cultus Lake sockeye salmon *(O. nerka)*, which uses the lake for adult spawning, embryo incubation and juvenile rearing prior to outmigration to the Pacific Ocean (COSEWIC, 2003). Water quality degradation is known to contribute to the risk of extirpation for this population, with eutrophication having the potential to disrupt the physical, chemical and biological aspects of its freshwater habitat (Putt *et al.*, 2019; DFO, 2020b).

As with most juveniles rearing in nursery lakes, Cultus Lake sockeye salmon undergo diel vertical migrations, holding in the deep, profundal lake waters during the day and ascending to shallower depths in the water column to feed through the crepuscular periods and at night (Levy, 1989; Scheuerell and Schindler, 2003). This behavioural cycle exposes them to multiple, spatially disparate water column stresses in Cultus Lake (DFO, 2020b). Late-season hypolimnetic DO concentrations have been reduced by more than half in Cultus Lake since the 1920s–1930s (Shortreed, 2007; Putt *et al.*, 2019), with the most pronounced depletion occurring in the lake profundal zone, associated with the decomposition of excess organic matter arising from the overlying water column under lake eutrophication (DFO, 2020b; Gauthier *et al.*, 2021). Juvenile sockeye salmon are sensitive to hypoxia (Brett, 1964; Davis, 1975; Ruggerone, 2000) and rely upon sufficient levels of DO in hypolimnetic and profundal habitats, which presents a refuge from lake predators (Scheuerell and Schindler, 2003 In Cultus Lake, hydroacoustic data indicate most juveniles reside in close association with the lake sediments during the daylight hours, despite evident hypoxia. Elevated water temperatures in the upper water column (i.e. epilimnion, metalimnion) may further expose sockeye salmon juveniles to thermal stresses during foraging. Together, these stressors may kill or weaken fish and increase their susceptibility to disease and other stressors.

In this study, we sampled sockeye salmon juveniles using mid-water trawling at night, when the fish are feeding at shallower depths in the water column. Although sockeye salmon juveniles may not have been directly exposed to either hypoxic or thermal stressors at the time of sampling, the selected biomarkers are more highly activated under chronic than acute stresses (e.g. Jeffries *et al.*, 2014; Anttila *et al.*, 2015; Houde *et al.*, 2019; Akbarzadeh *et al.*, 2018, 2020), and thus should reflect the stress signatures of their recent environmental experiences. For instance, the induction of hypoxia and thermal responsive genes in fish exposed to either water temperature and hypoxia stresses remains upregulated hours after returning to CTs and normal oxygen (i.e. 6–8 h; Buckley *et al.*, 2006; Qi *et al.*, 2020). Using molecular biomarkers specific to hypoxic and thermal stresses independently, we have demonstrated that during summer and early fall months when Cultus Lake exhibits profundal hypoxia and high water temperatures in the epilimnion, over one-third of the fish sampled were responding to hypoxic and thermal stresses concurrently. Hypoxic stresses affected more fish during July and September than in the other months with HP conditions in Cultus Lake. Furthermore, thermal stresses affected more fish when epilimnetic water temperatures approached or exceeded 20°C. About 25.7% of fish sampled during months when both stressors were pronounced in Cultus Lake (July, September, October) exhibited transcriptomic signatures of both stressors. By applying both thermal and hypoxic stress biomarkers concurrently, we detected additive effects of each stressor in 92% of the fish sampled, when both environmental stressors were present, suggesting that for several months each year, most juvenile sockeye salmon in Cultus lake are impacted by these anthropogenically linked environmental stresses (e.g. eutrophication, climate change).

Our data confirm that sockeye salmon juveniles in Cultus Lake are exposed to and affected by both low DO water in profundal habitats and elevated temperatures in the upper water column, during the mid-to late-stratified period (i.e. summer, fall), as a consequence of the interplay between their natural diel vertical migrations and anthropogenic degradation of their critical freshwater habitat. Sockeye salmon juveniles migrate into warmer surface waters in lakes to forage upon available prey and then migrate into cooler deeper waters through the daylight period, most likely to take advantage of better food conversion efficiencies and to reduce piscivorous predation risks (Scheuerell and Schindler, 2003). Indeed, hypoxia appears to be a strong stressor on juvenile Cultus Lake sockeye salmon, particularly in summer and fall, when fish inhabit the deeper portions of the hypolimnion (i.e. profundal zone) during the daylight hours. Moreover, when fish move up into the shallower depths to feed at night, their transcriptomic signatures indicate they also encounter physiologically stressful water temperatures, inducing a serial and cumulative environmental stress context for the majority of these endangered salmonids. In the small percentage of juvenile sockeye salmon rearing in Cultus Lake, which exhibited no genomic evidence of neither stressor, during months when oxygen and thermal stresses co-occur (July–October), we speculate these fish may have spent less time in degraded deep and shallow waters, and it is possible that these fish simply behaviourally moderated the length of time spent in each habitat, reducing their risk of exposure to such chronic stresses. Such compensatory behaviour does not necessarily come without consequences, as it may diminish feeding opportunities and enhance predation rates (Scheuerell and Schindler, 2003).

Elevated temperatures and hypoxia are considered to have synergistic effects, meaning that a small change in one stressor could cause a large change in the capacity of animals to respond to either of the stressors when the animals are simultaneously exposed (McBryan *et al.*, 2013). The probability of synergistic effects of hypoxia and temperature on whole-animal physiology follows from observations that the maximum oxygen consumption of tissues increases with temperature more than the capacity of the circulatory system to supply oxygen to tissues, leading to anaerobic energy production at higher temperatures (Anttila *et al.*, 2015). Sub-optimal DO concentrations can negatively impact growth in sockeye salmon juveniles (Brett and Blackburn, 1981). Avoidance of hypoxic bottom waters can reduce or eliminate low-temperature thermal refuges for organisms and increase energy demands and respiration rates, potentially reducing overall fitness, if alternative habitats are also sub-optimal (Roman *et al.*, 2019). Hypoxic conditions can reduce egg and fry survival and growth, eliciting larger population-level impacts (Del Rio *et al.*, 2019). Our results confirm that both lake water warming and hypoxia are important factors to consider in conservation mitigation strategies for sockeye salmon and other species at risk in Cultus Lake, the Cultus Lake Pygmy Sculpin (*Cottus aleuticus*, Cultus Population), which was recently uplisted to endangered by the Committee on the Status of Endangered Wildlife in Canada (COSEWIC, 2019) and for which eutrophication and profundal habitat degradation from declining DO are primary threats (DFO, 2017). We surmise that the additive impacts of chronic stresses observed in over 90% of fish sampled during peak water temperatures in summer likely impact fish condition, ecological performance, susceptibility to disease and ultimately survival. Sockeye salmon in Chilliwack Lake also exhibited signals of thermal stresses in the summer (August), when the water temperature was upwards of 20°C. However, the highly oxic and abundantly cool hypolimnion of Chilliwack Lake may permit physiological recovery from thermal stresses experienced in warm months. Sockeye salmon in Chilliwack Lake do not show genomic signatures of cumulative impacts of hypoxic and thermal stressors, which could contribute to their higher freshwater survival (COSEWIC, 2017).

The detection rate for the pathogen *I. multifiliis* was higher in Cultus Lake than in Chilliwack Lake; however, the genomic-inferred prevalence of this ciliate varied considerably among sampling months. The parasitic ciliate *I. multifiliis* commonly infects salmonid fry and parr in hatcheries causing serious outbreaks of disease (Valtonen and Keränen, 1981; Rintamäki-Kinnunen and Valtonen, 1997); it has also been observed in wild smolts beginning their migration to the ocean (Nekouei *et al.*, 2018). The disease caused by *I. multifiliis* is commonly known as ‘ich’ or ‘white spot disease’ because of the visible, raised white cysts in the epithelium of infected fish. Severe infections of the parasite in gill epithelia result in the loss of the respiratory, excretory and osmoregulatory functions of this organ, eventually leading to the death of the host (Traxler *et al.*, 1998). Epizootics of *I. multifiliis* occurred in adult pre-spawning and spawning sockeye salmon during the 1994 and 1995 spawning seasons in the Skeena River watershed in northern British Columbia, Canada (Traxler *et al.*, 1998). Heavy infections of *I. multifiliis* can certainly cause high mortality in salmonids, including wild stocks of sockeye salmon (Kent, 2011). The rate of development and maturation of *I. multifiliis* is positively related to temperature (Alhua and Buchmann, 2001). However, our data did not show a correlation between the prevalence genomic signatures of *I. multifiliis* and temperature in sockeye salmon juveniles inhabiting Cultus and Chilliwack lakes. While in Chilliwack Lake, the inferred prevalence was substantially higher in the warm month of August than the cool fall period, in Cultus Lake, two of the three peaks in inferred prevalence were observed in cooler periods with normoxia. The exception was in September when the parasite peaked at 96.4% of fish in Cultus Lake. Interestingly, we found that during the cool fall period in November, marked by profundal hypoxia, fish in Cultus Lake exhibited a remarkably higher prevalence of *I. multifiliis* than the normoxic Chilliwack Lake. This may indicate a relationship between the prevalence of *I. multifiliis* and hypoxic stresses in fish, although there is no established relationship in the literature.

Hypolimnetic oxygen depletion, a consequence of nutrient loading and increased deepwater aerobic decomposition of the resulting organic matter in Cultus Lake has been identified as a threat to the persistence of the local sockeye salmon population. The degradation of deep water habitat through oxygen depletion, coupled with warming epilimnetic water in the summer months may force juvenile sockeye salmon to forage and hold in suboptimal conditions that may be deleterious to their growth and survival. Therefore, strategies that mitigate eutrophication are needed for the conservation of this species. The nutrient loadings to Cultus Lake are known to be from multiple sources both whithin the lake watershed (i.e. subwatershed runoff, migratory gull guano and septic leachate) and the regional airshed (i.e. atmospheric deposition from intensive regional agriculture, transportation corridors and urban development) (Putt, 2014). Eutrophication mitigation strategies should therefore include controlling nutrient inputs to Cultus Lake from both the immediate watershed and the broader regional airshed. At the watershed level, eliminating the septic leachate, reducing agricultural runoff and the use of non-lethal tactics to reduce the migratory gull numbers, and consequently their guano, could be implemented to reduce the current nutrient loading in Cultus lake (Putt, 2014). Moreover, a multifaceted approach that targets airshed sources, i.e. atmospheric deposition within the lower mainland of British Columbia is also required to halt or reverse eutrofication trends (Putt, 2014; Putt *et al.*, 2019).

## Conclusion

In summary, using the curated molecular biomarker panels on Salmon Fit-Chips and limnological monitoring, we found that the majority of endangered sockeye salmon rearing in Cultus Lake showed molecular signals of exposure to hypoxic and thermal stress during summer and fall months. The stress caused by hypoxia and high temperature in warmer seasons, together with the high prevalence of the highly pathogenic ciliate, *I. multifiliis*, likely contribute to increased mortality of sockeye salmon juveniles in Cultus Lake. It is highly likely that lake eutrophication and hypolimnetic oxygen depletion are leading to increased risk of extirpation for sockeye salmon in Cultus Lake, and possibly the Cultus Pygmy Sculpin, which requires oxic hypolimnetic and profundal habitats. Thus, mitigation of the various identified and quantified nutrient loadings to Cultus Lake, particularly in the watershed and regional airshed, is crucial to restoring deep-water oxic habitats to promote conservation of this species, particularly under climate change (Putt *et al.*, 2019; DFO, 2020b; Gauthier *et al.*, 2021).

## Funding

Funding for this research was provided by the Pacific Salmon Commission Southern Endowment Fund [grant no. 2018-EF-043].

## Conflicts of interest

The authors declare no financial and personal conflict of interest.

## Appendix 1: Gene expression profile of 11 hypoxia and 10 thermal biomarkers for Sockeye salmon in Cultus and Chilliwack lakes from July 2018 to April 2019.

Hypoxia biomarkers:

Thermal bilomarkers:
